# Genome-Wide Identification of the *MdKNOX* Gene Family and Characterization of Its Transcriptional Regulation in *Malus domestica*

**DOI:** 10.3389/fpls.2020.00128

**Published:** 2020-02-21

**Authors:** Peng Jia, Chenguang Zhang, Libo Xing, Youmei Li, Kamran Shah, Xiya Zuo, Dong Zhang, Na An, Mingyu Han, Xiaolin Ren

**Affiliations:** ^1^ College of Horticulture, Northwest Agriculture and Forestry University, Yangling, China; ^2^ College of Life Sciences, Northwest Agriculture and Forestry University, Yangling, China

**Keywords:** *Malus domestica*, Knotted1-like Homeobox, flower induction, *growth-regulating factor*, yeast one-hybrid

## Abstract

Knotted1-like Homeobox (KNOX) proteins play important roles in regulating plant growth, development, and other biological processes. However, little information is available on the *KNOX* gene family in apple (*Malus domestica* Borkh.). In this study, 22 *KNOX* genes were identified in the apple genome. The gene structure, protein characteristics, and promoter region were characterized. The *MdKNOX* family members were divided into three classes based on their phylogenetic relationships. Quantitative real-time PCR analysis revealed that the majority of *MdKNOX* genes exhibited strongly preferential expression in buds and were significantly up-regulated during the flower induction period. The transcript levels of *MdKNOX* genes were responsive to treatments with flowering- and stress-related hormones. The putative upstream regulation factor *MdGRF* could directly bind to the promoter of *MdKNOX15* and *MdKNOX19*, and inhibit their transcriptional activities, which were confirmed by yeast one-hybrid and dual-luciferase assays. The results provide an important foundation for future analysis of the regulation and functions of the *MdKNOX* gene family.

## Introduction

Homeobox proteins are considered to act as sequence-specific DNA-binding proteins and contain a 60 amino-acid-long DNA-binding domain termed a homeodomain (HD) that directly regulates the expression of specific groups of target genes (Hayashi and Scott, 1990). Different HD proteins have been grouped into separate families (or classes) based on either sequence identity within the HD or conserved protein motifs outside of the HD (Bürglin and Affolter, 2016). Although their structures are similar, different homeodomains are able to recognize diverse DNA binding sites (Berger et al., 2008). *KNOTTED1-LIKE HOMEOBOX* (*KNOX*) genes belong to the three amino acid loop extension (TALE) homeodomain superfamily and are generally distinguished by four characteristic domains—KNOXI, KNOXII, ELK, and HD—although some genes lack the ELK and HD domains (Gao et al., 2015). The first homeobox gene reported in a plant species was *ZmKN1* from maize (Vollbrecht et al., 1991). Subsequently, a number of KNOX proteins have been characterized in many plant species (Hay and Tsiantis, 2010). Arabidopsis KNOX proteins can be divided into Classes I and II based on sequence similarity conventionally. Further, KNOX lost the HD domain was found in Arabidopsis, which defined a novel class, named as the KNATM (Magnani and Hake, 2008). Four Class I (*SHOOT-MERISTEMLESS (STM)*, *KNAT1*, *KNAT2*, and *KNAT6*) and Four Class II *KNOX* (*KNAT3*, *KNAT4*, *KNAT5,* and *KNAT7*) genes were identified from *Arabidopsis*. Class I genes have been intensively studied and shown to play important roles in meristem maintenance, control of leaf blade shape, internode elongation, hormone homeostasis, and establishment of inflorescence architecture (Tsuda et al., 2011; Tsuda and Hake, 2015). Loss-of-function mutations in the *Arabidopsis STM* resulted in embryos that lack a SAM (Barton and Poethig, 1993). *KNAT1* transcripts are detected in whole-shoot and inflorescence tissue but not in leaves, and *KNAT2* transcripts are present at high levels in shoot and inflorescence tissue as expected but are of low abundance in leaves, which affects leaf morphological development (Byrne et al., 2000). *KNAT6* is expressed at the site of lateral root initiation, and is involved in meristem activity and organ separation (BellesBoix et al., 2006). With regard to Class II *KNOX* genes, expression patterns have been characterized in maize by RNA gel-blot analysis (Kerstetter et al., 1994). Serikawa et al. (1997) detected *Arabidopsis KNAT3* expression patterns through the use of promoter-GUS (β-glucuronidase) fusion analysis and *in situ* hybridization. The varied expression patterns indicate that *KNAT3* plays several different roles in plants, depending on when and where it is expressed. Despite several reports of expression patterns, comparatively little is known about the function of Class II *KNOX* genes in plants. In *Arabidopsis*, domain exchange and phenotypes analysis suggest that the sequences outside of the third helix and N-terminal arm of the homeodomain endow the specificity of KNAT3 and KNAT1 (Serikawa and Zambryski, 1997). The Class II genes *KNAT3*, *KNAT4* and *KNAT5* perform redundant and important functions in root (Truernit and Haseloff, 2007) and lateral organ differentiation (Furumizu et al., 2015). Promoter-GUS and fluorescent protein analysis have demonstrated the transcriptional regulation and protein products localization of *KNAT3*, *KNAT4*, and *KNAT5* in specific domains and cell types of the *Arabidopsis* root (Truernit et al., 2006). *KNAT3* may also modulate abscisic acid (ABA) responses to regulate germination and early seedling development (Kim et al., 2013). *KNAT3* and *KNAT7* are involved in secondary cell wall biosynthesis in *Arabidopsis* and *Populus* (Li et al., 2012; Wang et al., 2020) and *GhKNL1* participates in the regulation of fiber development in cotton (Gong et al., 2014). Three Class II *KNOX* genes, *MtKNAT3/4/5-like*, from *Medicago truncatula* regulate legume nodule boundaries and shape development (Di Giacomo et al., 2017).

Additional research has revealed that *KNOX* genes are involved in diverse developmental processes mainly by affecting the metabolism and signaling pathway of hormones (Chan et al., 1998; Himmelbach et al., 2002; Bolduc and Hake, 2009). *KNOX* genes activate cytokinin biosynthesis (Jasinski et al., 2005; Yanai et al., 2005). For example, in *M. truncatula*, *MtKNOX3* activates the cytokinin biosynthesis *ISOPENTENYL TRANSFERASE* (*IPT*) genes, regulates nodule development, and activates cytokinin biosynthesis upon nodulation (Azarakhsh et al., 2015). KNOX proteins have been reported to repress the production of gibberellins (GAs). On the one hand, *KNOX* negatively modulates the accumulation of GAs by controlling the abundance of *GA2-oxidase*, by binding to an intron of *ga2ox1* and up-regulating the metabolic gene (Bolduc and Hake, 2009). On the other hand, *KNOX* inhibits GA biosynthesis by down-regulation of the key biosynthetic gene *GA20-oxidase* (Kusaba et al., 1998; Rosin et al., 2003). In addition, KNOX proteins are involved in other hormonal signaling pathways. *KNOX* change the abundance of proteins associated with auxin transporter signaling components to regulate abscission in tomato (Ma et al., 2015). Rice *HOMEOBOX 1* (*OSH1*) represses the brassinosteroid phytohormone pathway through activation of brassinosteroid catabolism genes (*CYP734A2*, *CYP734A4*, and *CYP734A6*) and then arrests the growth of the SAM (Tsuda et al., 2014). KNAT3 interacts with a BELL-like homeodomain (BLH) protein and synergistically modulates ABA responses during germination and early seedling development in *Arabidopsis* (Dachan et al., 2013).

In addition to being a transcriptional regulator, *KNOX* genes are regulated by other protein factors to prevent misexpression. *Arabidopsis* BELL-like homeodomain proteins BLH2/SAW1 and BLH4/SAW2 act redundantly to regulate expression of one or more *KNOX* genes and to establish leaf shape (Kumar et al., 2007). *NtSVP*, a MADS-box transcription factor from tobacco, acts as a repressor of the *BP*-like Class I *KNOX* gene *NtBPL* by directly binding to the *NtBPL* promoter, causing shortened pedicels (Wang et al., 2015). YABBY contributes to the repression of *KNOX* genes (*STM*, *KNAT1*/*BP* and *KNAT2*) to prevent development of ectopic meristems in *Arabidopsis* (Kumaran et al., 2002). The *Arabidopsis* polycomb group (PcG) protein FERTILIZATION-INDEPENDENT ENDOSPERM (FIE) and CURLY LEAF (CLF) could also repress expression of *KNOX* genes (Katz et al., 2004). In particular, transcription of *KNOX* genes is indicated to be suppressed by a growth-regulating factor (GRF), and such interactions have been confirmed in several species, including barley (Osnato et al., 2010), *Arabidopsis* and rice (Kuijt et al., 2014). This result implies that the *GRF*–*KNOX* regulatory module was relatively conservative.

Although roles of *KNOX* genes in plant development have been partly elucidated in *Arabidopsis* and other species, little information is available about the possible roles of these genes in fruit crops. Apple (*Malus domestica*) is one of the most widely cultivated fruit trees. To date, only two studies have been reported concerning *KNOX* genes from apple (Watillon et al., 1997; Gao et al., 2015). Here, we conducted the genome-wide identification of members of the *KNOX* gene family in *M. domestica*. The expression profiles in various tissues and in response to exogenous hormone treatment as well as during the floral development period were explored. In addition, the regulatory interaction between *MdKNOX* and the putative upstream regulator *MdGRF* was tested. This study may provide a foundation for further investigation of the regulation and functions of the *MdKNOX* gene family.

## Materials and Methods

### Identification of KNOX Encoding Genes in the Apple (*Malus domestica* Borkh.) Genome

*Arabidopsis thaliana* and *Oryza sativa KNOX* protein sequences were downloaded from The *Arabidopsis* Information Resource (TAIR, https://www.arabidopsis.org/) and the Rice Genome Annotation Project (http://rice.plantbiology.msu.edu/cgi-bin/ORF_infopage.cgi) databases. To identify the genes encoding KNOX proteins in the apple (*M. domestica*) genome, the BLASTP program was used to search for potential KNOX-encoding genes in the complete genome, using the known KNOX sequences from *Arabidopsis* and rice as queries. All non-redundant putative protein sequences were manually checked with the Pfam database (http://pfam.xfam.org/search/sequence) and the NCBI Conserved Domains database (https://www.ncbi.nlm.nih.gov/Structure/cdd/wrpsb.cgi). The 22 *MdKNOX* genes obtained were designated *MdKNOX1* to *MdKNOX22* based on their chromosomal locations. Protein physicochemical characteristics were predicted with the ExPASy program (http://web.expasy. org/protparam/).

### Multiple Sequence Alignment and Phylogenetic Analysis

The full-length amino acid sequences of KNOX proteins from *Arabidopsis*, rice, and apple were used for multiple alignment performed with DNAMAN software. We chose the following parameter settings—substitution matrix: Blosum62, mismatch score: −15, and gap open/extend penalty: 10/5. Phylogenetic trees were constructed using the MEGA 7.0 program (Kumar et al., 2016). Sequence alignment was carried out using MUSCLE (Edgar, 2004) program with default parameters. The optimal protein substitution model was the Jones–Taylor–Thornton (JTT) model with gamma distribution. The evolutionary history was inferred using the neighbor-joining method based on the JTT matrix-based model and gamma distribution. Support for the phylogeny topology was assessed by means of a bootstrap analysis with 500 replications. Sequence logos were generated using the Weblogo online platform (http://weblogo.berkeley.edu/logo.cgi).

### Gene Structure, Conserved Motif, and Promoter Sequence Analysis

A gene structures map was obtained with the Gene Structure Display Server (http://gsds.cbi.pku.edu.cn). Conserved motifs in MdKNOX protein sequences were elucidated with the MEME platform (http://meme-suite.org/) (Bailey et al., 2006). The dimensional structure of MdKNOX proteins was predicted with the PHYRE server v2.0 (http://www.sbg.bio.ic.ac.uk/phyre2/html/page.cgi?id=index). The 1,500-bp genomic DNA sequence upstream of the start codon (ATG) of each *MdKNOX* gene was obtained from the apple genome sequence. *Cis*-elements in the promoters were identified using the PlantCARE database (http://bioinformatics.psb.ugent.be/webtools/plantcare/html/).

### Homologous Gene Pairs and Synteny Analysis

Information on syntenic blocks within the apple genome and between the Arabidopsis and apple genomes were downloaded from the Plant Genome Duplication Database (http://chibba.agtec.uga.edu/duplication/). The genome sequences of homologous KNOX proteins from Arabidopsis and apple were assessed using BLASTP. The OrthoMCL algorithm was used to identify paralogous genes within the apple genome as well as between the apple and Arabidopsis genomes (Li et al., 2003). The MCScan algorithm (Wang et al., 2012) was applied to detect syntenic blocks containing apple *KNOX* genes. Circos (Krzywinski and Schein, 2009) was used to visualize the syntenic relationships between the genomes.

### Plant Materials and Treatments

Samples were collected from six-year-old apple Fuji/T337/*Malus robusta* Rehd. trees for tissue-specific expression analysis, comprising roots, stems, leaves, buds, flowers, and fruits. Newly developed lateral roots of 1–2 mm diameter, stems of 2–3 mm diameter near to the shoot apices, fully expanded leaves adjacent to buds, flower buds, flowers at anthesis, and young fruits were collected, immediately frozen in liquid nitrogen, and stored at −80°C until use.

For hormone treatments, apple trees of uniform growth in the experimental orchard of the College of Horticulture, Northwest A&F University, Yangling, China (108°04′ E, 34°16′ N) were chosen and randomly divided into six groups. Each group was treated with 4 mmol/L salicylic acid (SA), 150 μmol/L ABA, 700 mg/L gibberellin (GA_3_), 300 mg/L 6-Benzylaminopurine (6-BA) or water (control). Solutions were applied by spraying the leaves with a low-pressure hand-wand sprayer, followed by sampling at 30, 50, and 70 days after full bloom (DAFB). Plant samples were immediately frozen in liquid nitrogen after collection and stored at −80°C until use.

### RNA Extraction, cDNA Synthesis, and Quantitative Real-Time PCR

Total RNA was isolated using a RNA extraction kit (OMEGA, Doraville, GA, USA). RNA integrity was verified by electrophoresis and RNA concentration was determined using a Nanodrop 2000 spectrophotometer. One microgram of total RNA was used as the template for first-strand cDNA synthesis, using the PrimeScript™ RT Reagent kit (Takara, Shiga, Japan) following the manufacturer's instructions.

Primer pairs for quantitative real-time PCR (qRT-PCR) were designed using Primer Premier 6.0 (Premier Biosoft, Palo Alto, CA, USA) (**Supplementary Table 1**). It was difficult to distinguish the amplification products because of the high similarity in coding regions among several *MdKNOX* genes. Therefore, the same primer pair was used to analyze the expression of both *MdKNOX1* and *MdKNOX20*, *MdKNOX2* and *MdKNOX5*, *MdKNOX4* and *MdKNOX12*, *MdKNOX10* and *MdKNOX22*. Consequently, 18 pairs of primers were designed for 22 *MdKNOX* genes. Each primer pair was checked *via* RT-PCR followed by 1.2% agarose gel electrophoresis to verify the specificity of the amplification products.

Real-time RT-PCR was performed in a total volume of 20 μl containing 2 μl cDNA, 10 μl of 2×SYBR^®^ Green II Mix, 0.5 μM of each primer, and distilled deionized H_2_O. Analyses were conducted with the Bio-Rad CFX Connect™ Real-Time PCR Detection System (Bio-Rad, Hercules, CA, USA). The PCR protocol was as follows: 94°C pre-incubation for 3 min; followed by 40 cycles of denaturation at 94°C for 10 s, and annealing at 60°C for 30 s. At the end of the amplification, a melting curve from 65 to 95°C with 0.5°C increments was performed to verify the presence of gene-specific PCR products. Apple *Actin* (MD04G1127400) and *HistoneH3* (MD01G1035300) were used as internal standard genes. Three biological replicates for each sample and three technical replicates for each biological replicate were analyzed. The relative expression levels were calculated using the relative 2^−△△^ *^Ct^* method (Livak and Schmittgen, 2001).

### Yeast One-Hybrid (Y1H) and Dual-Luciferase Assays

To clone the promoters of *MdKNOX15* and *MdKNOX19*, genomic DNA was isolated from fresh young leaves of apple ‘Fuji' and used as the DNA template. Each PCR system contained the Phusion^®^ High-Fidelity PCR Master Mix, 0.5 μM primer pairs (proKNOX15-F/proKNOX15-R for *MdKNOX15* promoter, and proKNOX19-F/proKNOX19-R for *MdKNOX19* promoter) and 1 ng/μl genomic DNA. The standard thermal profile was as follows: 95°C for 2 min; 30 cycles of 95°C for 10 s, 57°C for 20 s, and 72°C for 1 min; followed by a final extension for 10 min at 72°C. The PCR products were cloned into the pBlunt vector (CloneSmart, USA) for sequencing.

A yeast one-hybrid (Y1H) assay was performed using the Gold Matchmaker^™^ Gold Yeast One-Hybrid System (Clontech, Mountain View, CA, USA). The open reading frame of *MdGRF* was cloned using the primers MdGRF-F1 and MdGRF-R1, and inserted into the pGADT7 vector. Then the 277-bp or 434-bp promoter fragment of the *MdKNOX* gene (*proKNOX*) was inserted into the pAbAi vector. After linearization, the constructs were transformed into the yeast cells, which were plated on SD/−Ura media supplemented with aureobasidin A (AbA) to determine the minimal inhibitory concentration of AbA. Growth of the co-transformant yeast cells (harboring pGADT7-MdGRF and pAbAi-proMdKNOX) was detected on SD/−Ura medium supplemented with AbA.

For dual-luciferase assays, the complete expression units of improved firefly (coleopteran) luciferase (FLuc) and *Renilla* (*Renilla reniformis*) luciferase (RLuc) were cloned from pGL3 basic-2X35S-Rluc-2X35S-Fluc plasmid (Gu et al., 2013), and inserted into the multiple cloning site (MCS) of pCAMBIA0309 vector to generate the dual reporter expression vector. To detect the effect of *MdGRF* on the promoter activities of *MdKNOX*, the 2× 35S promoter upstream of the luciferase gene was replaced by the promoter of the *KNOX* gene to generate the reporter, and the open reading frame of *MdGRF* was cloned using the primers MdGRF-F2/MdGRF-R2 and inserted into pRI101-AN vector o generate the effector (MdGRF-OE) plasmid. The recombinant vectors were transformed into *Agrobacterium* strain GV3103. Tobacco leaves were infected with the mixed *Agrobacterium* cells by means of *Agrobacterium*-mediated transient injection (Krenek et al., 2015). RLuc/FLuc activity was assessed using the Dual-Luciferase^®^ Reporter Assay System (Promega, USA).

### Statistical Analysis

Data were subjected to analysis of variance and the means were compared using Student's *t*-test at the 5% significance level using SPSS 11.5 software (SPSS, Chicago, IL, USA).

## Results

### Genome-Wide Identification of *Arabidopsis* and Apple *KNOX* Genes

Nine *KNOX* genes were previously identified and reported in the *A. thaliana* genome, named *KNATM*, *KNAT6*, *STM*, *KNAT7*, *KNAT2*, *KNAT1*/*BP*, *KNAT5*, *KNAT4,* and *KNAT3*. To identify apple *KNOX* genes, a BLASTP search of the apple genome was conducted with the nine AtKNOX protein sequences as queries. After manual checking and confirmation using the NCBI Conserved Domains database, 22 candidate *MdKNOX* genes were obtained (**Table 1**). The *MdKNOX* genes were named in accordance with their chromosomal locations (*MdKNOX1*–*MdKNOX22*). The 22 *MdKNOX* genes were located on 12 chromosomes in the apple genome. The chromosomes 6 and 15 harbored the highest number of genes (three genes each), the chromosomes 4, 8, 10, 14 and 15 contained two genes, and chromosome 3, 5, 9,12, 16, and 17 each carried a single gene (**Table 1**).

**Table 1:** *Arabidopsis thaliana* and apple *KNOX* gene families.

Name	Gene ID	Location	CDS(bp)	Peptide(aa)
KNATM	AT1G14760	chr1:5,084,315..5,084,315	429	142
STM	AT1G62360	chr1:23,058,582..23,058,582	1,149	382
KNAT1/BP	AT4G08150	chr4:5,147,699..5,147,699	1,197	398
KNAT2	AT1G70510	chr1:26,576,486..26,576,486	933	310
KNAT6	AT1G23380	chr1:8,297,241..8,297,241	990	329
KNAT3	AT5G25220	chr5:8,735,944..8,735,944	1,296	431
KNAT4	AT5G11060	chr5:3,509,833..3,509,833	1,182	393
KNAT5	AT4G32040	chr4:15,493,989..15,493,989	1,152	383
KNAT7	AT1G62990	chr1:23,337,167..23,337,167	876	291
KNOX01	MD02G1012900	Chr03:821,764..821,764	1,083	360
KNOX02	MD04G1069700	Chr04:9,546,107..9,546,107	765	254
KNOX03	MD04G1215500	Chr04:29,846,452..29,846,452	993	330
KNOX04	MD05G1352500	Chr05:46,891,824..46,891,824	1,161	386
KNOX05	MD06G1071100	Chr06:17,202,997..17,202,997	867	288
KNOX06	MD06G1171700	Chr06:31,218,154..31,218,154	1,056	351
KNOX07	MD06G1232400	Chr06:36,328,062..36,328,062	426	141
KNOX08	MD08G1075200	Chr08:6,111,898..6,111,898	750	249
KNOX09	MD08G1153600	Chr08:16,709,140..16,709,140	1,293	430
KNOX10	MD09G1112500	Chr09:8,548,362..8,548,362	1005	334
KNOX11	MD10G1276200	Chr10:36,731,509..36,731,509	621	206
KNOX12	MD10G1326500	Chr10:40,664,274..40,664,274	1,182	393
KNOX13	MD12G1205700	Chr12:28,622,790..28,622,790	990	329
KNOX14	MD13G1018900	Chr13:1,182,375..1,182,375	330	109
KNOX15	MD13G1095800	Chr13:6,760,799..6,760,799	1,059	352
KNOX16	MD14G1177200	Chr14:27,054,900..27,054,900	933	310
KNOX17	MD14G1239200	Chr14:31,827,706..31,827,706	426	141
KNOX18	MD15G1062700	Chr15:4,315,813..4,315,813	753	250
KNOX19	MD15G1130800	Chr15:9,443,279..9,443,279	1,314	437
KNOX20	MD15G1159800	Chr15:11,950,827..11,950,827	1,107	368
KNOX21	MD16G1097200	Chr16:6,783,377..6,783,377	1,077	358
KNOX22	MD17G1102600	Chr17:8,723,554..8,723,554	987	328

**Figure 1:**
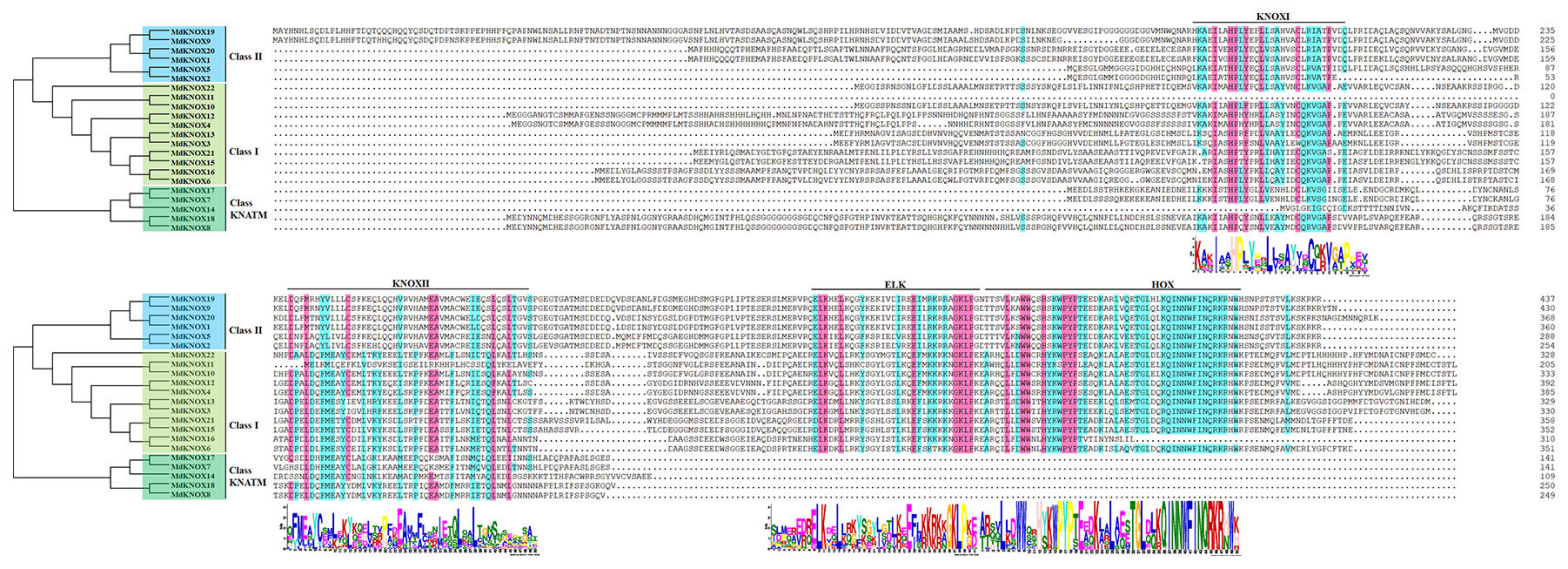
Multiple sequences alignment of MdKNOX proteins.

Multiple sequence alignment showed that the majority of the MdKNOX proteins shared four conserved domains: KNOXI, KNOXII, ELK, and HOX domain (**Figure 1**). The ELK and HOX domains were located at the C terminus of the MdKNOX protein, whereas the KNOXI and KNOXII domains were located at the N terminus. Among the proteins, ELK and HOX domains were absent in five MdKNOX proteins (MdKNOX7, MdKNOX8, MdKNOX14, MdKNOX17, and MdKNOX18) (**Figure 1**).

### Gene Characterization and Structure Analysis of *MdKNOX*

**Figure 2:**
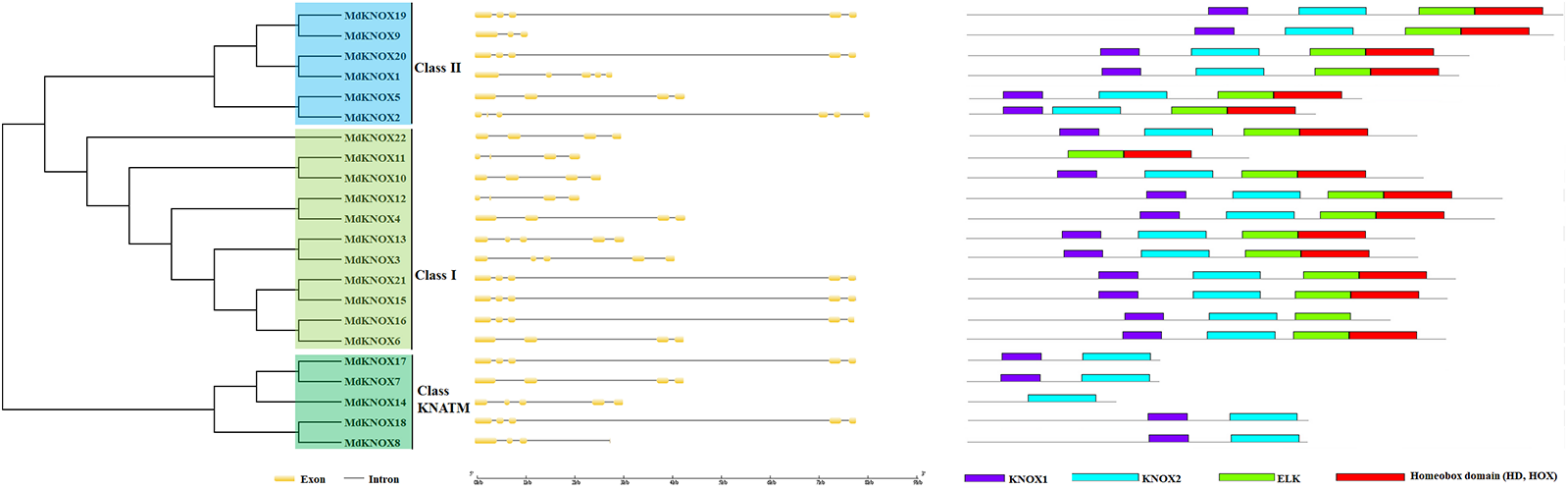
Analysis of *MdKNOX* gene structure. An unrooted neighbor-joining tree was constructed derived from MdKNOX protein sequences (left) and exon–intron composition analysis (right).

KNOX protein characteristics were analyzed using the ExPASy portal, including molecular weight, isoelectric point, grand average of hydropathicity, instability index, major amino acid content, and aliphatic index (**Table 2**). The molecular weight of the analyzed MdKNOX proteins ranged from 11.99 (MdKNOX14) to 40.81 kDa (MdKNOX1). The molecular weight of the MdKNOX proteins was greater than 13 except for MdKNOX14, which indicated that MdKNOX is a group of macromolecular proteins. The isoelectric point ranged from 4.66 (MdKNOX7) to 6.89 (MdKNOX11) (**Table 2**). Given that the instability index values were greater than 40, all KNOX proteins were considered to be unstable except for KNOX7. Grand average of hydropathicity values indicated that the MdKNOX proteins were hydrophilic. The aliphatic index values ranged from 54.15 (MdKNOX4) to 85.18 (MdKNOX17). Amino acid content analysis showed that Lys and Ser were the predominant residues, and that Glu, Ala, Gln, and Lys also accounted for a large proportion of the proteins. Alpha helices, β sheets, extended strands, and random coils were present in the predicted protein structures of all the MdKNOX proteins except for MdKNOX16 (**Supplementary Figure 1**). The Gene Structure Display Server was used to display the exon–intron structure based on the annotated apple genome. All *MdKNOX* family members contained 3–6 introns. The number and distribution of introns for *MdKNOX* genes was rather conserved within each cluster (**Figure 2**). For example, the *KNAT2/6* cluster, including *MdKNOX15*, *MdKNOX16*, and *MdKNOX21*, was highly conserved and comprised four introns and five exons. However, although the genes *MdKNOX2* and *MdKNOX5* showed high similarity in protein sequences, the distribution and location of exons were distinct. These differences suggested that the two genes have functionally diverged during evolution.

**Table 2:** Amino acid compositions as well as physical and chemical characteristics of KNOX proteins.

Name	pI	MW	Instability Index	GRAVY	Major Amino Acid	Aliphatic Index
MdKNOX01	5.19	40.81	53.81	−0.747	S(9.4%)L(9.4%)E(8.6%)D(7.5%)	72.03
MdKNOX02	6.27	29.08	58.41	−0.729	L(10.2%)E(9.0%)S(7.4%)G(7.0%)	74.45
MdKNOX03	5.14	36.83	41.83	−0.547	G(10.3%)E(9.6%)S(8.7%)L(8.4%)	67.36
MdKNOX04	6.3	43.04	44.81	−0.726	S(11.9%)G(7.5%)L(6.9%)A(6.7%)	54.15
MdKNOX05	6.31	32.85	59.96	−0.739	L(10.7%)Q(9.0%)S(8.3%)E(7.9%)	76.18
MdKNOX06	5.15	39.57	46.5	−0.636	S(10.2%)L(7.9%)A(7.4%)D(7.1%)	65.38
MdKNOX07	4.66	15.73	36.75	−0.655	L(13.4%)E(11.3%)K(8.5%)S(8.5%)	83.05
MdKNOX08	5.87	27.70	49.68	−0.895	S(9.6%)N(9.2%)G(8.8%)Q(7.6%)	57.19
MdKNOX09	5.95	48.31	47.72	−0.788	L(9.3%)S(8.8%)A(7.2%)N(7.2%)	71.02
MdKNOX10	6.32	37.72	48.13	−0.61	L(10.1%)S(9.8%)A(7.4%)E(7.1%)	68.71
MdKNOX11	6.89	24.08	45.14	−0.723	L(11.6%)K(9.7%)E(7.7%)S(7.2%)	71.99
MdKNOX12	6.32	43.91	48.22	−0.734	S(11.1%)L(7.6%)A(7.1%)G(6.8%)	56.41
MdKNOX13	5.14	36.82	41.95	−0.663	S(9.4%)E(9.1%)G(9.1%)L(8.2%)	63.74
MdKNOX14	4.88	12.00	40.56	−0.492	S(9.1%)A(8.2%)D(8.2%)K(8.2%)	64.5
MdKNOX15	5.17	39.87	46.5	−0.679	S(10.7%)L(8.8%)E(8.5%)D(7.1%)	65.2
MdKNOX16	4.94	34.65	50.12	−0.553	S(10.9%)L(8.7%)A(8.3%)D(7.0%)	67.45
MdKNOX17	4.72	15.78	49.08	−0.561	L(12.7%)E(11.3%)S(9.2%)K(7.8%)	85.18
MdKNOX18	5.85	27.74	46.13	−0.857	S(10.0%)G(9.2%)N(9.2%)Q(7.6%)	57.76
MdKNOX19	6.01	48.66	49.41	−0.762	S(8.9%)L(8.6%)A(7.3%)Q(7.0%)	68.99
MdKNOX20	5.43	41.65	47	−0.799	S(8.9%)L(8.6%)D(7.8%)E(7.6%)	68.61
MdKNOX21	5.62	40.88	43.57	−0.643	S(10.3%)L(8.9%)E(7.8%)A(7.2%)	67.37
MdKNOX22	6.16	37.11	47.42	−0.564	L(10.3%)S(10.3%)A(7.9%)E(7.3%)	71.46
KNATM	5.64	16.44	56.91	−0.349	L(14.0%)S(11.2%)K(9.1%)E(8.4%)	87.89
STM	6.19	42.75	55.48	−0.652	S(12.0%)A(7.8%)L(7.0%)E(6.8%)	55.99
KNAT1/BP	6.02	45.84	50.08	−1.113	N(10.5%)S(10.0%)L(7.5%)E(7.2%)	56.41
KNAT2	4.9	35.64	48.97	−0.705	D(9.6%)L(9.6%)S(8.3%)E(8.0%)	70.87
KNAT6	4.92	37.19	53.6	−0.542	S(10.0%)L(9.1%)D(8.5%)E(8.5%)	73.56
KNAT3	5.86	47.60	57.27	−0.696	A(9.2%)L(8.8%)S(8.5%)Q(7.6%)	69.56
KNAT4	5.87	44.39	67.02	−0.856	S(9.6%)L(8.9%)E(8.3%)Q(8.3%)	66.82
KNAT5	6.03	43.28	55.48	−0.651	L(9.3%)S(9.3%)E(7.5%)T(6.7%)	73.79
KNAT7	6.1	32.91	47.98	−0.61	L(10.3%)E(8.2%)A(7.2%)G(6.8%)	76.43

pI, isoelectric point; MW, molecular weight; kDa, GRAVY: grand average of hydropathicity.

### Synteny Analysis and Phylogenetic Relationships Among *KNOX* Genes

**Figure 3:**
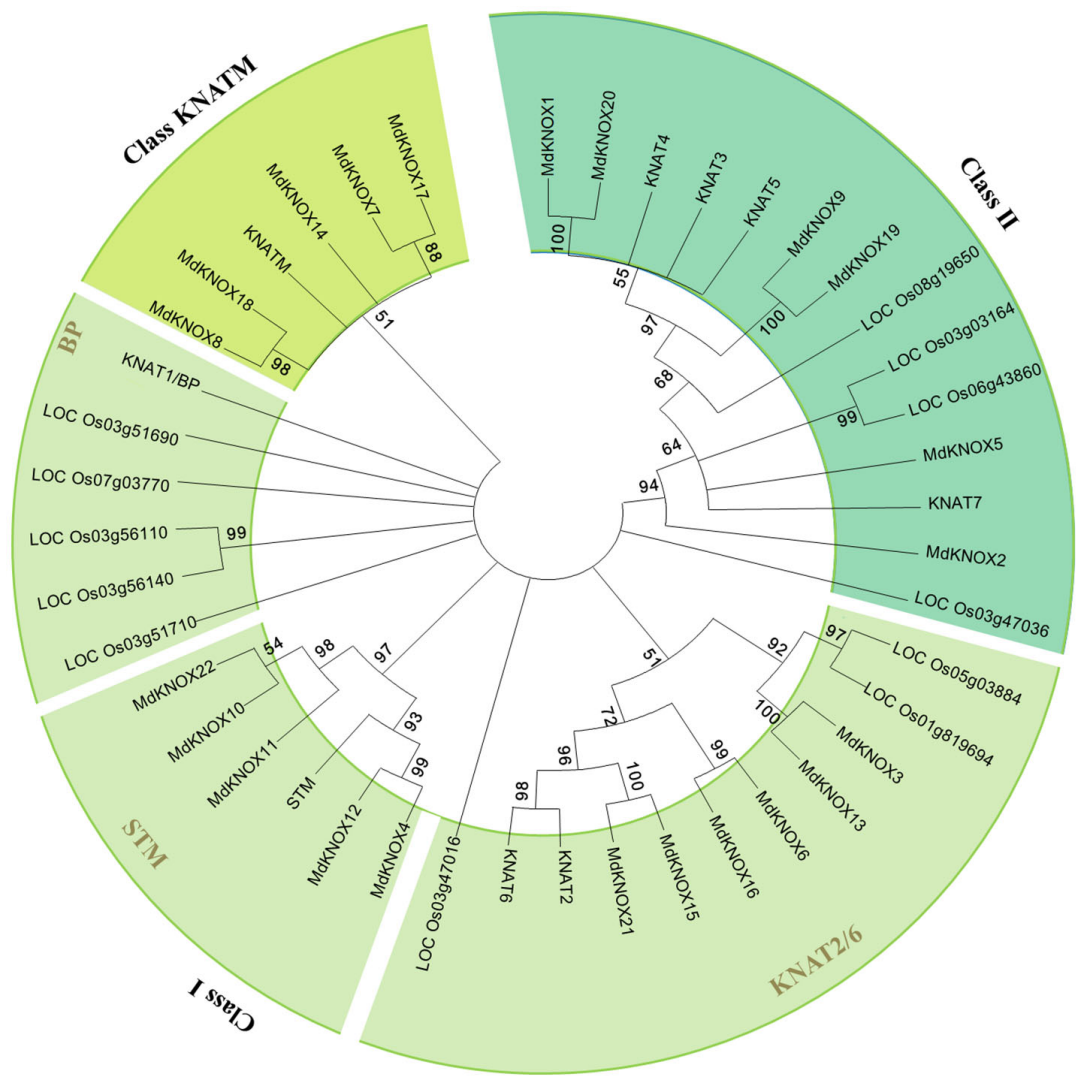
Neighbor-joining tree representing phylogenetic relationships among *KNOX* genes from apple, Arabidopsis, and rice.

To clarify the evolutionary relationships among *KNOX proteins*, a neighbor-joining tree was constructed derived from *Arabidopsis, rice,* and apple KNOX protein sequences. According to the phylogenetic tree (**Figure 3**), the *KNOX* proteins were clustered into three groups, designated Class I, Class II and Class KNATM. Class I was further divided into four subgroups: STM, KNAT2, KNAT6, and BP. Six apple proteins (MdKNOX1, MdKNOX2, MdKNOX5, MdKNOX9, MdKNOX19, and MdKNOX20) were clustered in Class II, and eleven apple proteins were clustered in the Class I. MdKNOX7, MdKNOX8, MdKNOX14, MdKNOX17, and MdKNOX18 were clustered in Class KNATM (**Figure 3**), which lakes the HOX domain (**Figure 1**)

**Figure 4:**
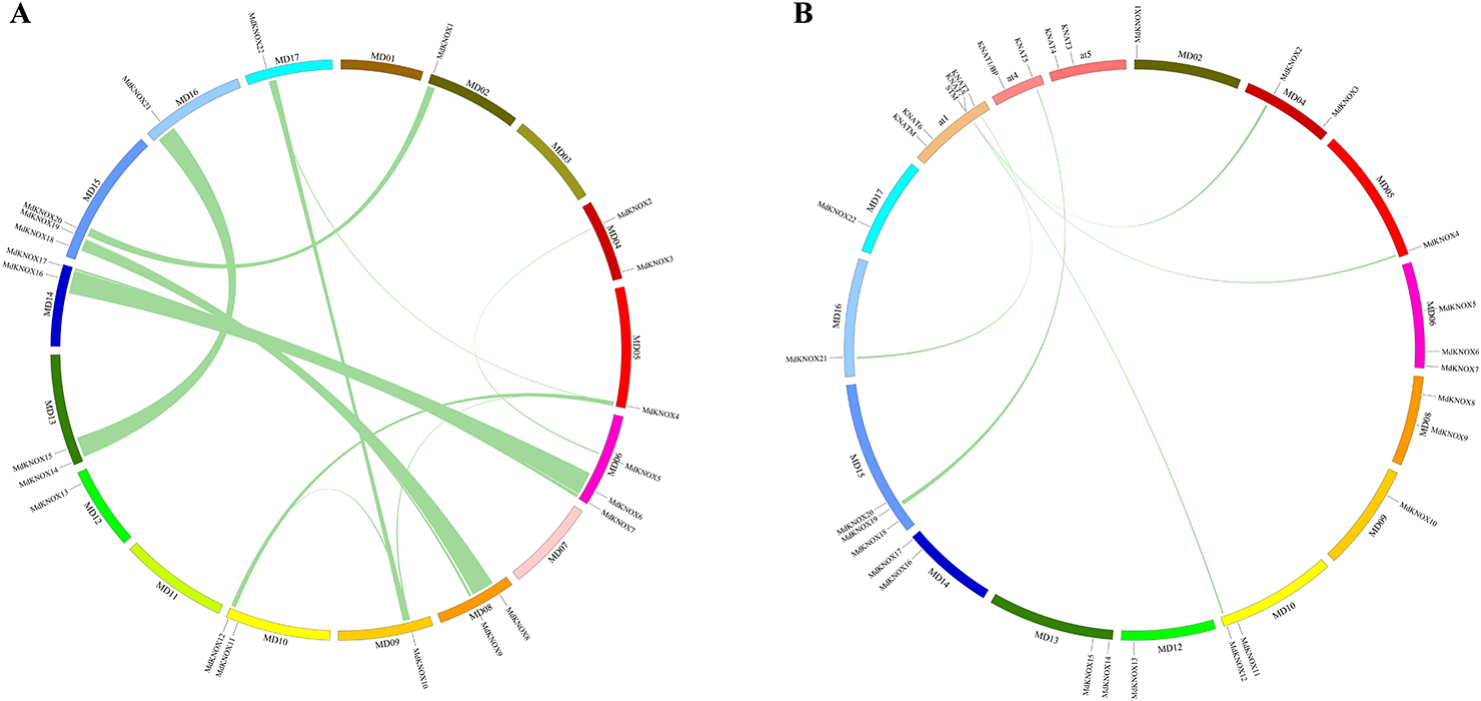
Analysis of evolutionary relationships among *KNOX* gene family members. Relative positive positions are depicted according to apple chromosomes, colored lines indicate syntenic regions of the apple genome **(A)**. Synteny analysis of *KNOX* genes between Arabidopsis and apple; relative positive positions are depicted according to apple and *Arabidopsis* chromosomes, colored lines indicate syntenic regions of the apple and *Arabidopsis* genomes **(B)**.

Segmental and tandem duplications are reported to be the predominant mechanisms of diversification of the *KNOX* gene family (Cannon et al., 2004). To analyze *MdKNOX* gene duplication events, the Circos software was used to detect duplicated blocks in the apple genome. More than ten pairs of *MdKNOX* genes, such as *MdKNOX1*/*MdKNOX20*, *MdKNOX2*/*MdKNOX5*, *MdKNOX4*/*MdKNOX12*, *MdKNOX7*/*MdKNOX17*, *MdKNOX8*/*MdKNOX18*, *MdKNOX10*/*MdKNOX22*, *MdKNOX12*/*MdKNOX21*, were located in duplicated genomic regions. Chromosomes 1, 3, 5, 7, and 12 did not contain any duplicated genes, whereas Chromosomes 6 and 15 contained the highest number of duplications (**Figure 4A**). Given that *Arabidopsis* is a well-characterized model plant species, we generated a comparative *KNOX* synteny map between *Arabidopsis* and apple to investigate orthologous genes and extract information on evolutionary relationships between the two species. Five pairs of syntenic orthologous genes were matched between the two species, including *KNAT5*/*MdKNOX19*, *KNAT2*/*MdKNOX21*, *STM*/*MdKNOX12*, *STM*/*MdKNOX4*, and *KNAT7*/*MdKNOX2* (**Figure 4B**).

### *MdKNOX* Expression Patterns in Different Tissues

**Figure 5:**
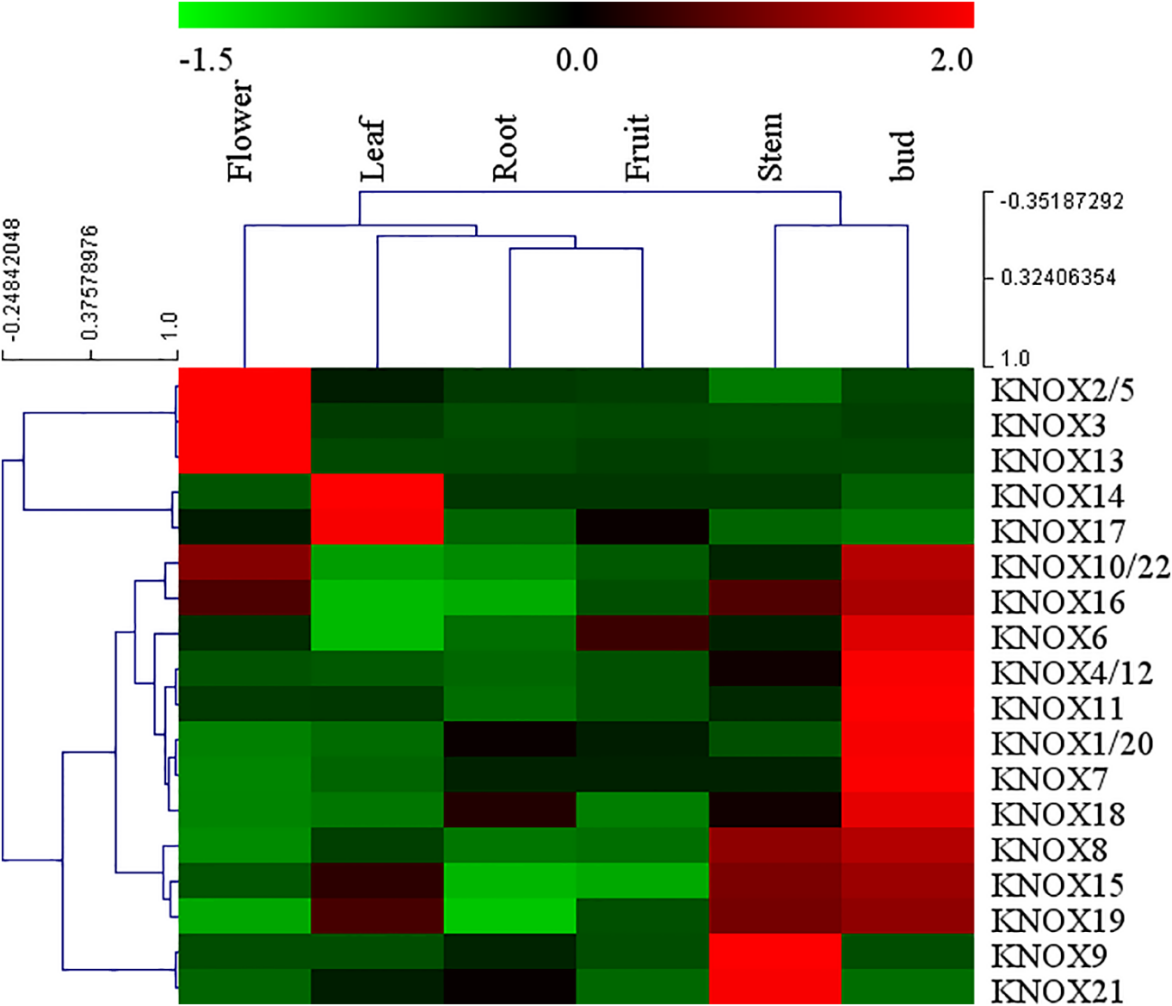
*MdKNOX* gene expression profiles in different tissues. The heat map was generated using MEV software. Relative expression profiles are based on quantitative real-time PCR data.

Arabidopsis *KNOX* genes have been well characterized, whereas little information on expression of apple *KNOX* genes is available. To elucidate the expression patterns of *MdKNOX* genes in apple, the expression patterns in a variety of tissues were analyzed by qRT-PCR. A heat map was drawn to visualize the expression profiles of individual *MdKNOX* genes based on the qRT-PCR data (**Figure 5**). The majority of (fourteen) *MdKNOX* genes exhibited strongly preferential expression in the floral bud. *MdKNOX8*, *MdKNOX15*, *MdKNOX16*, and *MdKNOX19* were highly expressed in the floral bud and stem. Low expression levels in the root and fruit were recorded for all *MdKNOX* genes except for *MdKNOX16* and *MdKNOX12*. Only *MdKNOX2*/*5*, *MdKNOX3*, *MdKNOX10*/*22*, *MdKNOX13*, and *MdKNOX16* were highly expressed in the flower, and *MdKNOX14*, *MdKNOX15*, *MdKNOX17*, and *MdKNOX19* were highly expressed in the leaf.

### *MdKNOX* Expression Patterns During the Flower Induction Period

**Figure 6:**
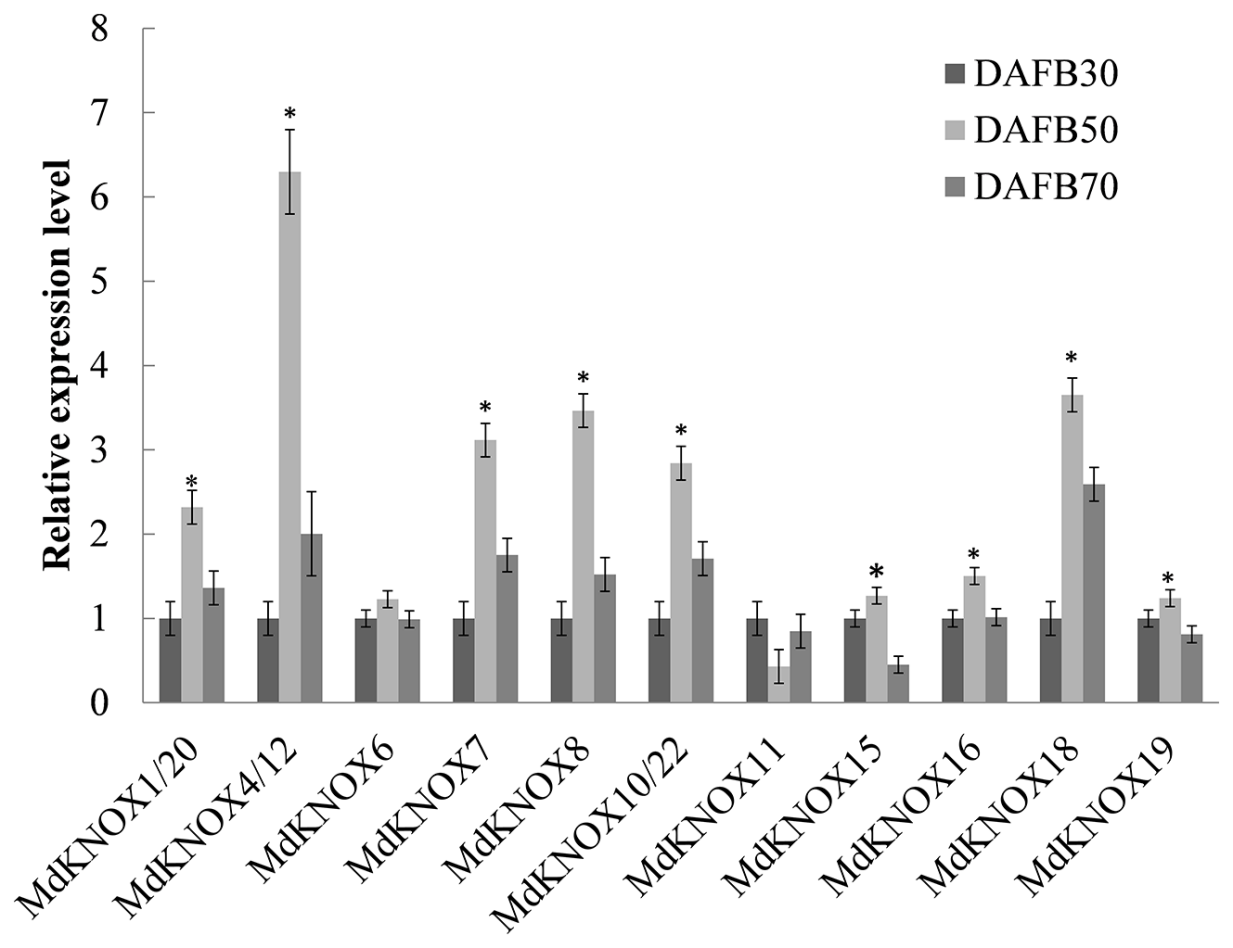
*MdKNOX* gene expression profiles during the flower induction period. Samples were collected at 30, 50, and 70, days after full bloom (DAFB). Each value represents the mean ± standard error of three replicates. Asterisks (*) means significant difference at the 0.05 level.

Fourteen *MdKNOX* genes that exhibited strongly preferential expression in the floral bud were chosen to detect *MdKNOX* expression patterns during the flower induction period (**Figure 6**). The transcript level of the majority of these *MdKNOX* genes was increased, including *MdKNOX1*/*20*, *MdKNOX4/12*, *MdKNOX7*, *MdKNOX8*, *MdKNOX10*/*22*, *MdKNOX15*, *MdKNOX18*, and *MdKNOX19*. However, the gene expression level of *MdKNOX6* was not significantly up-regulated and that of *MdKNOX11* showed a downward trend.

### Effect of Phytohormone Treatments on *MdKNOX* Expression During the Flower Induction Period

**Figure 7:**
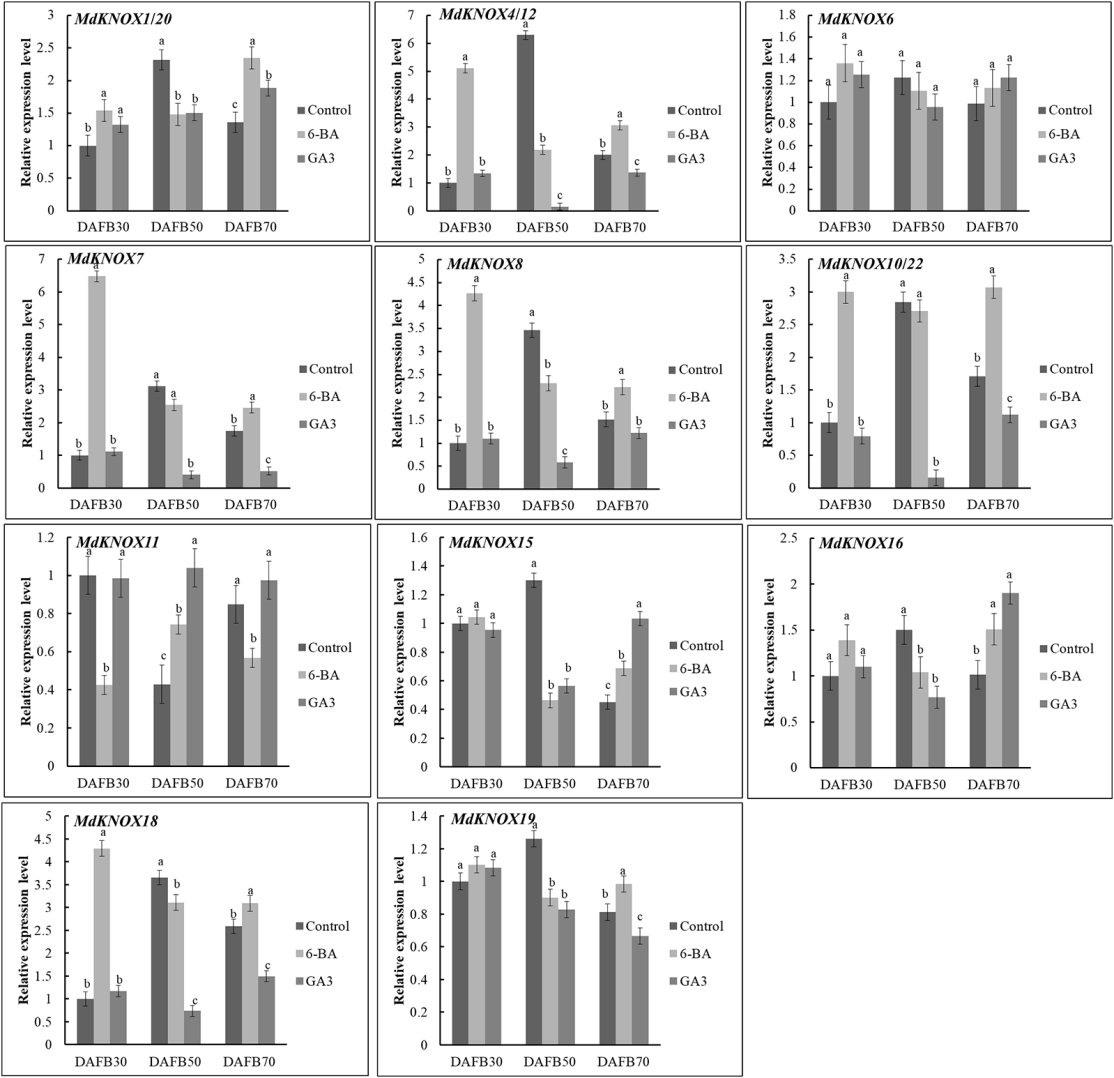
*MdKNOX* transcript levels in response to 6-benzylaminopurine (6-BA) and gibberellic acid (GA_3_) treatments. Samples were collected at 30, 50, 70 days after full bloom (DAFB) after 6-BA or GA_3_ treatment, with water used as a control. Each value represents the mean± standard error of three replicates. Different letters means significant difference at the 0.05 level.

**Figure 8:**
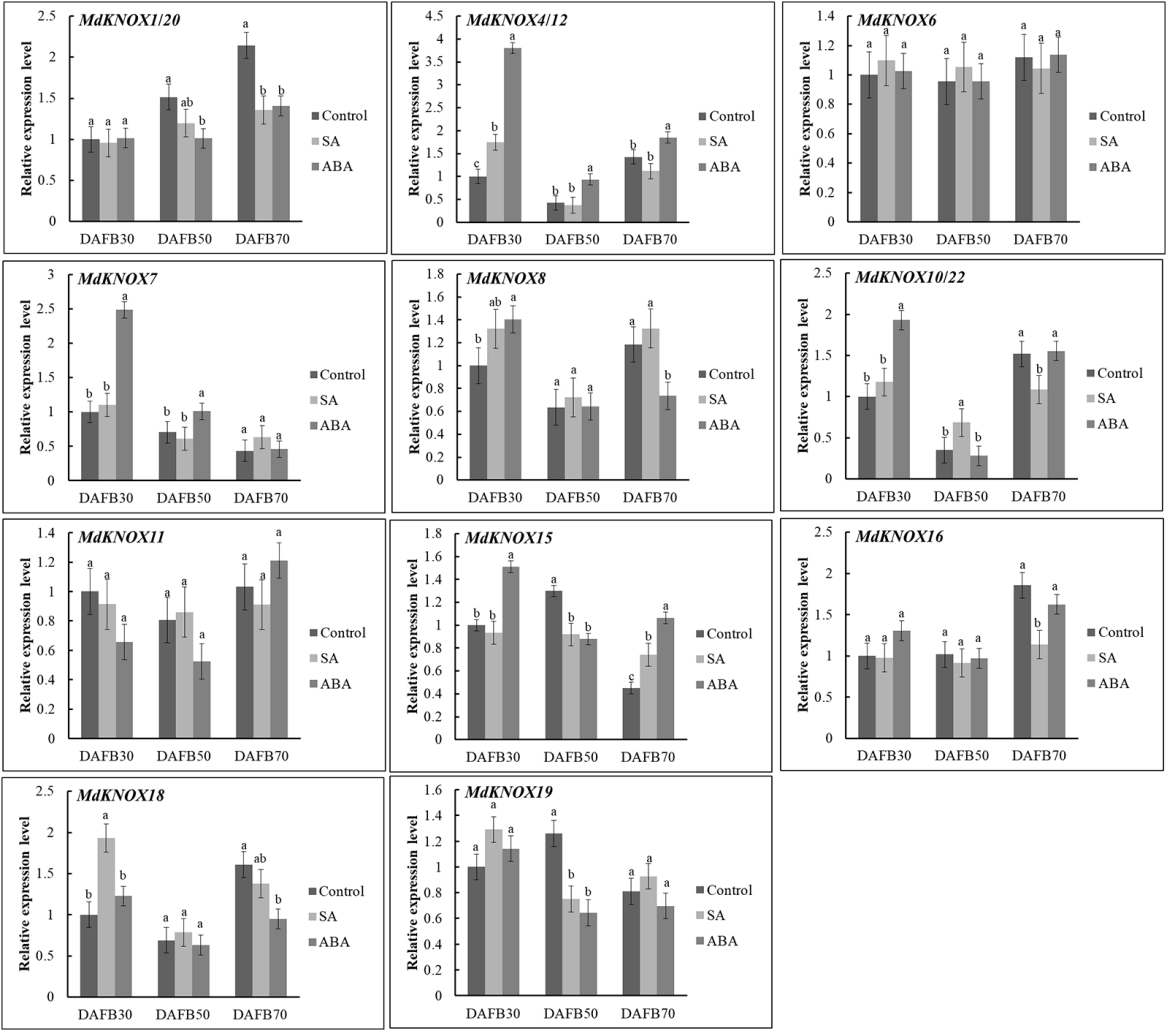
*MdKNOX* expression levels in response to SA and ABA treatments. Samples were collected at 30, 50, 70 days after full bloom (DAFB) after SA or ABA treatment, with water used as a control. Each value represents the mean ± standard error of three replicates. Different letters means significant difference at the 0.05 level.

To assess the potential effects of phytohormones on *MdKNOX* expression during the flower induction period, the transcript levels were estimated after treatment with 6-BA, GA_3_, ABA, or SA. Treatment with 6-BA significantly increased the transcript levels of *MdKNOX4/12*, *MdKNOX7*, *MdKNOX8*, *MdKNOX10/22*, and *MdKNOX18* at DAFB30 (**Figure 7**). At DAFB50, all *MdKNOX* genes showed lower transcript levels compared with that of the control except for *MdKNOX11*. *MdKNOX11* was down-regulated in response to 6-BA treatment in both the early and late sampling periods but was significantly induced at DAFB50. *MdKNOX1/20*, *MdKNOX4/12*, *MdKNOX7*, *MdKNOX8*, *MdKNOX10/22*, *MdKNOX15*, *MdKNOX16*, *MdKNOX18*, and *MdKNOX19* also showed higher transcript levels than that of the control at DAFB70. In response to exogenously applied GA_3_ (**Figure 7**), *MdKNOX* expression was unaffected at DAFB30, whereas all *MdKNOX* genes except *MdKNOX11* were down-regulated at DAFB50, especially *MdKNOX4/12*, *MdKNOX7*, *MdKNOX8*, *MdKNOX10/22*, and *MdKNOX18*. The transcript abundance of *MdKNOX4/12*, *MdKNOX7*, *MdKNOX8*, *MdKNOX10/22*, and *MdKNOX18* remained at lower levels than that of the control at DAFB70. *MdKNOX* expression patterns varied over time in response to SA and ABA treatment (**Figure 8**). *MdKNOX4/12*, *MdKNOX15*, and *MdKNOX18* were initially up-regulated in response to SA treatment and subsequently showed no significant difference at DAFB50 and DAFB70. *MdKNOX15* transcription was suppressed at DAFB50. *MdKNOX1/20* was significantly inhibited by SA at DAFB70 and *MdKNOX19* was significantly inhibited at DAFB50*. MdKNOX10/22* was up-regulated by SA at DAFB50, while down-regulated at DAFB70. With regard to ABA (**Figure 8**), *MdKNOX4/12*, *MdKNOX7*, *MdKNOX8*, *MdKNOX10/22*, *MdKNOX15*, and *MdKNOX16* were up-regulated at DAFB30. However, no significant difference was observed for all the *MdKNOX* at subsequent time points between control and ABA-treated group except for *MdKNOX15* and *MdKNOX19*.

### Analysis of the cis-Elements in the *MdKNOX* Promoters

**Figure 9:**
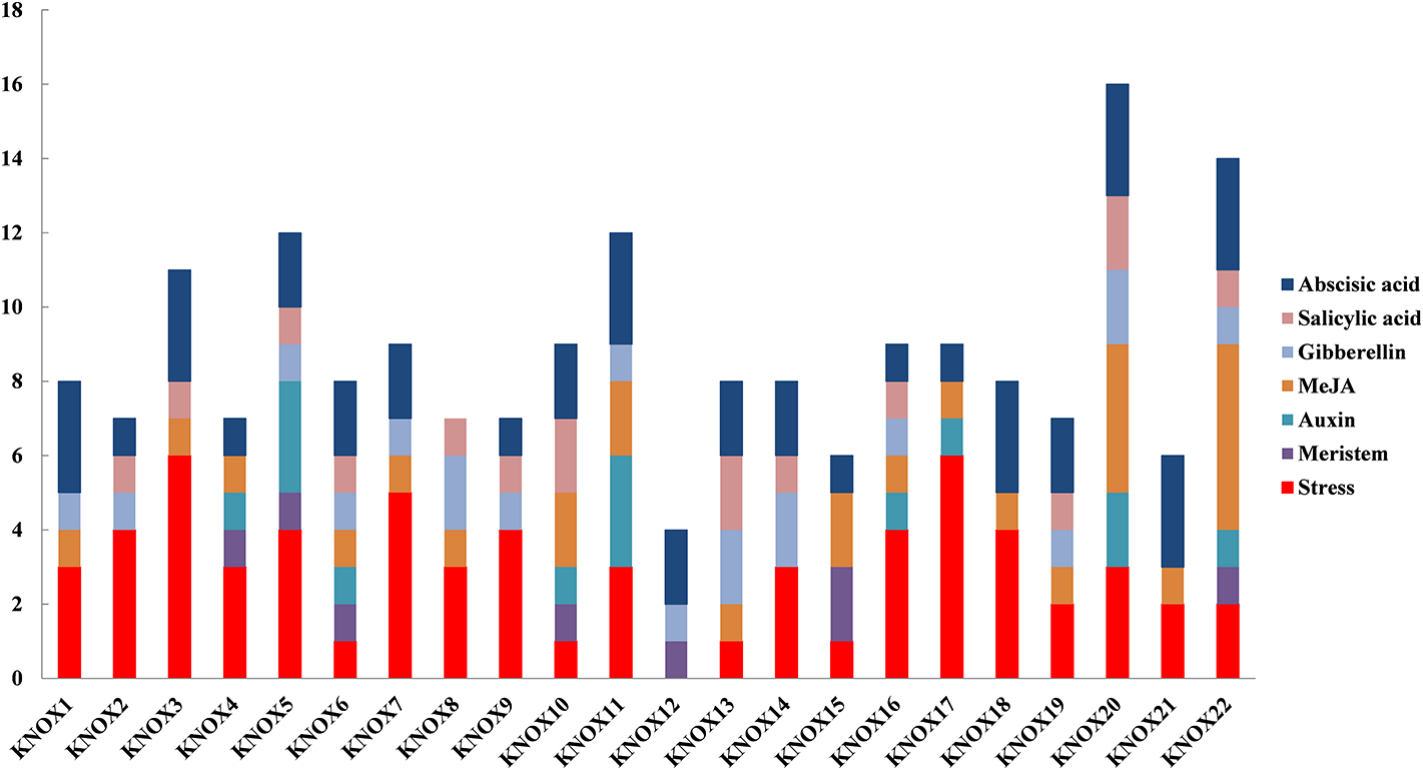
Predicted *cis*-elements in the *MdKNOX* promoters. The 1.5 kb sequence upstream from the start codon of *MdKNOX* genes was analyzed using the PlantCARE database.

To further investigate the regulatory mechanisms and potential functions of *MdKNOX* genes, *cis*-element motifs associated with responses to environmental factors and phytohormones were detected in the 1.5-kb promoter region upstream of the start codon (ATG) (**Figure 9**). Stress-related elements were detected in the promoters of all *MdKNOX* genes except for *MdKNOX2*. Meristem-related *cis*-elements were also identified in the *MdKNOX4*, *MdKNOX5*, *MdKNOX6*, *MdKNOX10, MdKNOX15,* and *MdKNOX22* promoters. Several hormone-related *cis*-elements were detected in all *MdKNOX* genes, including ABA-, SA-, GA-, methyl jasmonate-, and auxin-responsive elements. Among the *cis*-acting elements involved in hormone-related responses, the ABA-responsive element was present (as one to three copies) in all studied promoters except for *MdKNOX8*.

### MdGRF Directly Binds to the *MdKNOX15* and *KNOX19* Promoter

**Figure 10:**
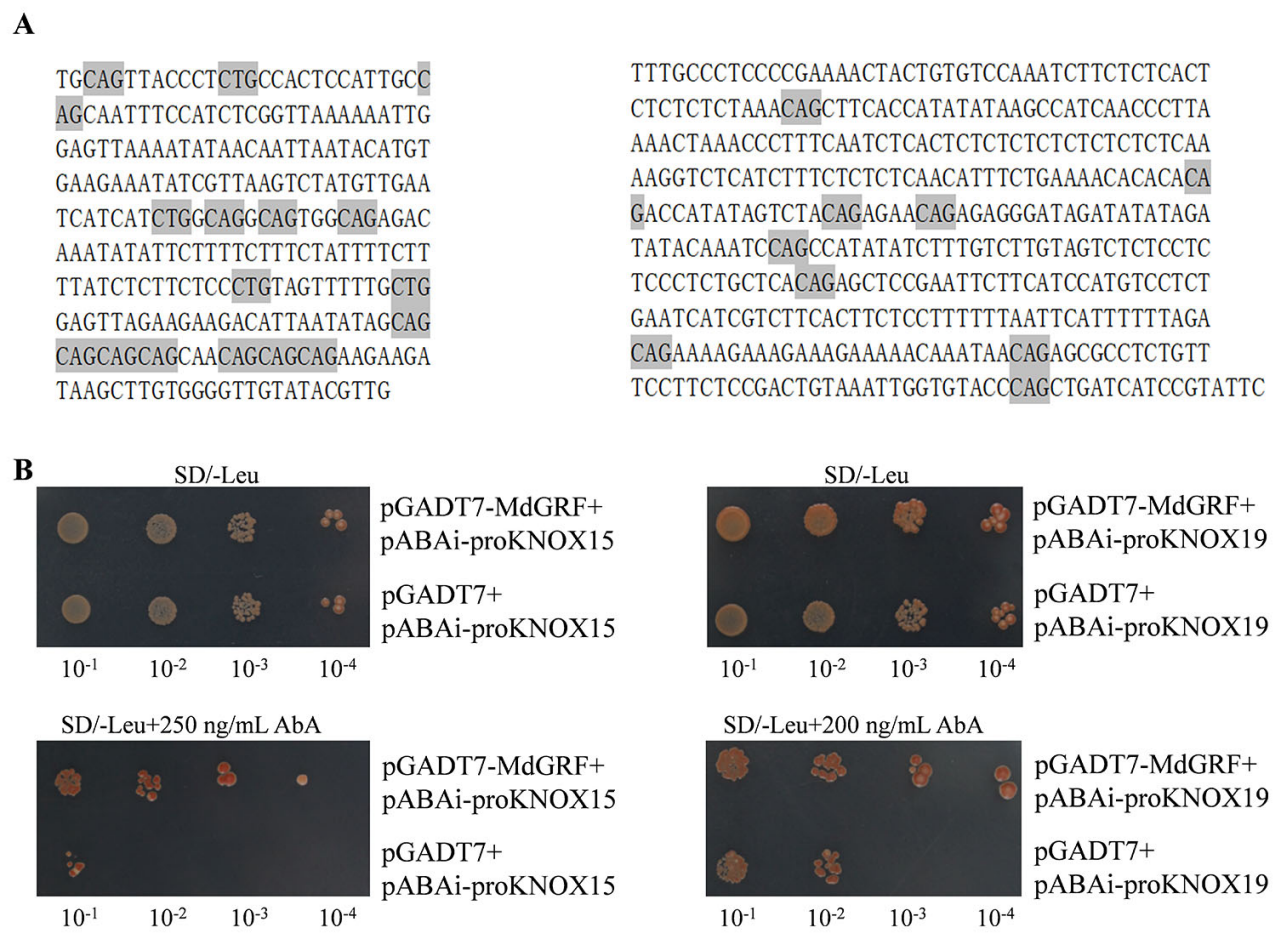
MdGRF binds to *MdKNOX15* and *MdKNOX19* promoters. **(A)** Promoter sequences of *MdKNOX15* and *MdKNOX19* genes used for yeast one-hybrid (Y1H) assay. The putative *GRF* binding sites, core CAG repeats or its reverse complementary sequence CTG were marked with gray shadow. **(B)** The yeast strains were grown on SD/-Leu and SD/-Leu/+ AbA medium for 3 d.

We conducted Y1H assays to test the interaction between the MdGRF protein and *MdKNOX15 and MdKNOX19* promoters. The open reading frame of MdGRF was cloned into pGADT7 vector. The promoter fragments of *MdKNOX15* and *MdKNOX19* (**Figure 10A**) were inserted into the pAbAi vector, respectively. Yeast strains carrying the pGADT7-MdGRF and pAbAi-proKNOX constructs grew normally on selective medium supplemented with AbA (200 ng/ml for *KNOX15* and 250 ng/ml for *KNOX19*), whereas the pGADT7 empty vector control did not grow (**Figure 10B**). These results suggested that *MdGRF* directly interacted with the *MdKNOX* promoter.

### MdGRF Inhibited the Promoter Activities of *MdKNOX15* and *MdKNOX19*

**Figure 11:**
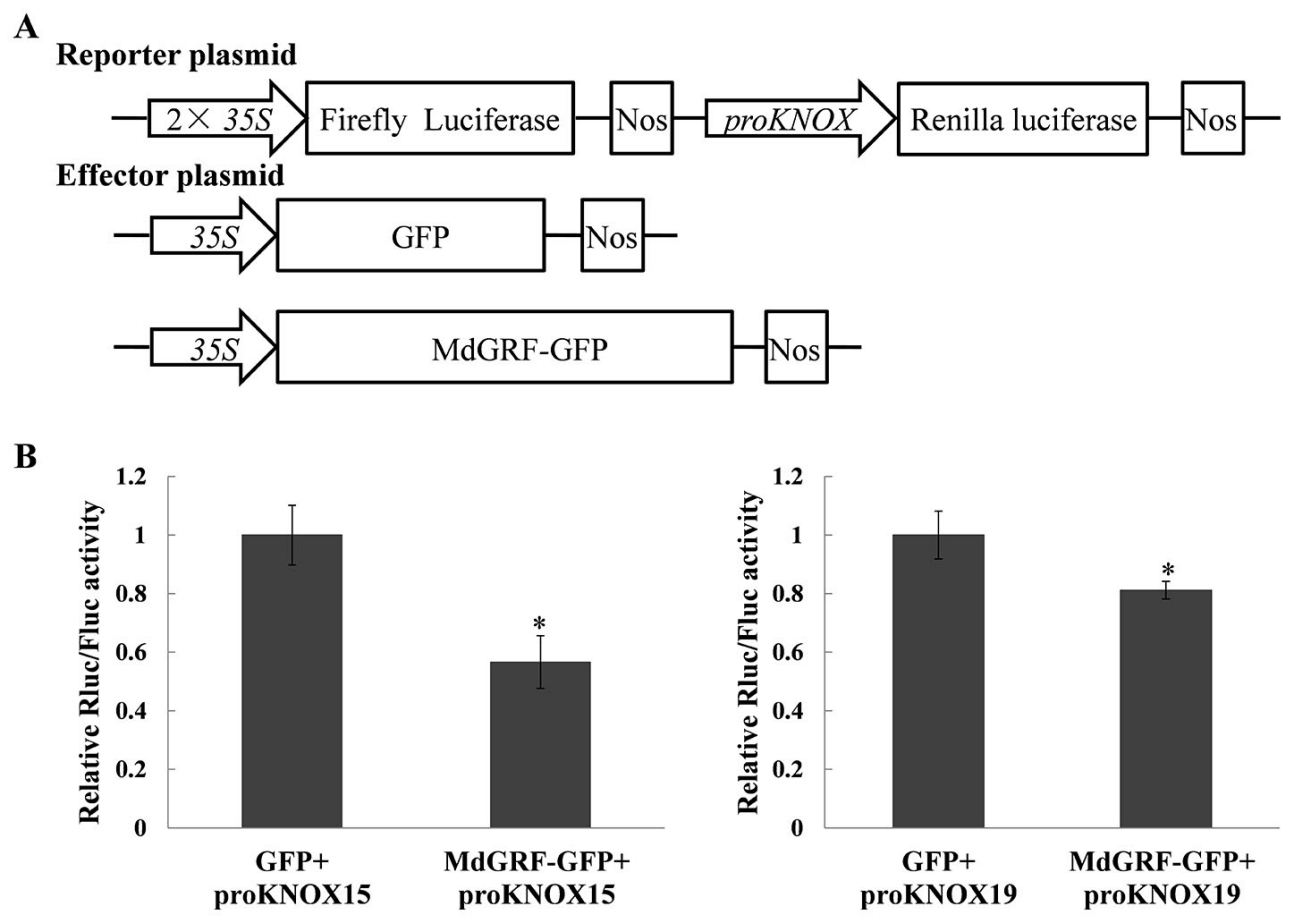
MdGRF inhibited the promoter activities of *MdKNOX15* and *MdKNOX19*. **(A)** Schematic diagram of the reporter vector and effector vector. **(B)** Dual-luciferase assays showing that MdGRF inhibits the transcription *MdKNOX15* and *MdKNOX19* in tobacco leaves. The MdGRF effector vector (MdGRF-GFP) or the control effector vector (GFP) with the reporter vector containing the *MdKNOX* promoter (proKNOX15 and proKNOX19) were infiltrated into tobacco leaves for analysis of Rluc/Fluc activity. Each experiment was performed in three replicates. Asterisks (*) indicates a significant difference (*p <*0.05) compared with the control.

To test whether MdGRF regulated the transcription of *MdKNOX* genes, a transient transformation assay was conducted. A dual effector–reporter system was established using *MdGRF* as the effector and the RLuc gene under the control of the *MdKNOX* promoter as the reporter (**Figure 11A**). The Rluc/Fluc activity was decreased under co-transformation with 35S:GRF-GFP and proKNOX compared with that under co-transformation with 35S:GFP and proKNOX (**Figure 11B**). These results suggested that MdGFR negatively regulated the expression level of *MdKNOX15* and *MdKNOX19*.

## Discussion

### Identification of Apple *KNOX* Genes

We identified 22 *MdKNOX* genes in the apple genome, which is greater than the number of *KNOX* genes identified in *Arabidopsis* and rice, and may reflect that the apple genome (881 Mb) is larger than those of rice (466 Mb) and *Arabidopsis* (12 Mb). The identified *MdKNOX* genes were unevenly distributed on 12 of the 17 apple chromosomes (**Table 1**).

Multiple sequence alignment showed that the majority of MdKNOX proteins contained a series of conserved domains: KNOXI, KNOXII, ELK, and HOX domains (**Figure 1**). The HOX domain is located in the C-terminal portion of the protein and is involved in DNA binding and possibly in homodimer formation (Scofield and Murray, 2006). The ELK domain is located adjacent to the HD domain, spans about 21 amino acids, and is composed of a conserved series of Glu (E), Leu (L), and Lys (K) amino acids. The ELK domain may function as a nuclear localization signal and also is considered to be involved in transcriptional repression, but the precise role of this domain has not been determined (Kerstetter et al., 1994; Sakamoto et al., 1999; Nagasaki et al., 2001). The KNOXI and KNOXII domains are located in the N-terminal half of the protein. KNOXI plays a role in suppressing target gene expression and KNOXII is considered to be necessary for homodimerization (Nagasaki et al., 2001) and transactivation (Scofield and Murray, 2006).

### Phylogenesis, Evolution, and Expansion of *MdKNOX* Gene

An unrooted neighbor-joining tree was constructed from a multiple alignment of the KNOX protein sequences from apple, rice, and *Arabidopsis* to investigate evolutionary relationships. The analysis separated the KNOX proteins into three groups. *MdKNOX1*, *MdKNOX2*, *KNOX5*, *MdKNOX9*, *MdKNOX19*, and *MdKNOX20* were clustered with *KNAT3*, *KNAT4*, *KNAT5*, and *KNAT7*, which belong to the Class II group (**Figure 3**). The majority of *MdKNOX* genes were clustered into the Class I group, which consisted of four subfamilies. MdKNOX7, MdKNOX8, MdKNOX14, MdKNOX17, and MdKNOX18 lost the ELK and HOX domains, which clustered in the Class KNATM (**Figure 1** and **3**). *KNOX* genes with similar functions and structural motifs showed a tendency to cluster in the same subgroup, which provided a foundation to explore the functions of each *MdKNOX* gene.

Previous research has shown that gene duplications are important in the evolution of species. Genome-wide duplication events occurred in apple about 60 million years ago, resulting in expansion from nine to 17 chromosomes and diversification of some gene families (Velasco et al., 2010). A number of apple gene duplications have been reported, such as the *CCOs* (Chen et al., 2018), *IDD* (Fan et al., 2017a), and *GASA* families (Fan et al., 2017b). In the present study, eight gene pairs were tentatively identified as duplicated genes (**Figure 4A**). Gene duplications and expansion resulted in *MdKNOX* gene clusters and increased the diversification of *MdKNOX* gene structures and functions.

Genomic comparisons with orthologous genes from well-studied plant species may provide a valuable reference for newly identified genes (Koonin, 2005). Thus, the functions of *MdKNOX* were inferred by comparative genomic analyses with the *KNOX* genes from *Arabidopsis*. Five orthologous gene pairs between *Arabidopsis* and apple were identified (**Figure 4B**), which suggested that the genes in question may share a common ancestor their functions have been conserved during evolution. Although many genetic prediction resources are available, additional research is needed to determine the specific function of each gene.

### *MdKNOX* Gene Expression Profiles and Potential Functions

Given the gene functional diversity, all members of the *MdKNOX* gene family need to be further functionally characterized. Analysis of tissue expression patterns of *MdKNOX* genes may provide insights into their possible functions. The majority of *MdKNOX* family members showed high transcript levels in floral buds, whereas extremely low transcript levels were detected in roots (**Figure 5**). We observed some differences between the present results and previous reports for other plant species that Class II *KNOX* genes in angiosperms are expressed in differentiating organs, including leaves, stems, flowers, and roots (Kerstetter et al., 1994). On the other hand, according to the more accurate *KNAT3* promoter-driven GUS staining patterns, the Class II *KNOX* gene *KNAT3* is highly expressed in cotyledons, and apical and floral tissues, and is moderately expressed in roots. Moreover, light has a significant effect on the expression profile of *KNAT3* (Serikawa et al., 1997). Therefore, we inferred that the developmental stage, sampling method, and species specificity may affect the experimental results. Despite these differences, detection of high transcript levels in floral buds implied that the majority of genes (*MdKNOX1*/*20*, *MdKNOX4*/*12*, *MdKNOX6*, *MdKNOX7*, *MdKNOX8*, *MdKNOX10*/*22*, *MdKNOX11*, *MdKNOX15*, *MdKNOX16*, *MdKNOX18*, and *MdKNOX19*) were involved in the regulation of flowering.

Insufficient production of flower buds is an intractable problem in the apple industry. The physiological differentiation of apple flower buds is essential for flowering and fruiting. Therefore, we analyzed the expression of the *MdKNOX* genes that were highly expressed in floral buds during the flower induction period (floral bud physiological differentiation, at DAFB30, DAFB50, and DAFB70). The genes were highly induced at 50 DAFB (**Figure 6**), suggesting that these genes may play an active role in floral induction. Several phytohormone-associated *cis*-element motifs were predicted within the *MdKNOX* promoters (**Figure 9**). In addition, we analyzed the expression profiles under different hormone treatments. The exogenous plant hormone 6-BA promotes flower bud formation (Li et al., 2019), whereas GA_3_ reduces flowering rates in apple (Zhang et al., 2016). *MdKNOX1/20* showed identical expression patterns in response to 6-BA and GA_3_ treatments. The transcription of *MdKNOX1/20* was strongly induced by both 6-BA and GA_3_ at DAFB30 and DAFB70, but was inhibited at DAFB50. *MdKNOX4/12* showed a similar expression pattern to that of *MdKNOX1/20* in response to 6-BA treatment. Treatment with 6-BA also increased the transcript levels of *MdKNOX4/12*, *MdKNOX7*, *MdKNOX8*, *MdKNOX10/22*, and *MdKNOX18* in the initial stage of flower induction (DAFB30), whereas transcription of the genes was suppressed at the intermediate stage of flower induction (DAFB50). Only *MdKNOX11* was down-regulated by 6-BA (in the early and late sampling stages), but was induced at 50 DAFB. With regard to *MdKNOX15*, *MdKNOX16*, and *MdKNOX19*, 6-BA treatment affected their expression at 30 DAFB, and each gene was suppressed at 50 DAFB by 6-BA. This finding is similar to previously reported results, for example, *KNAT3* transcript levels are decreased in response to exposure to kinetin (Truernit et al., 2006). These results implied that *MdKNOX* genes were regulated by 6-BA and might also regulate the 6-BA hormone signal. All *MdKNOX* genes did not show a significant difference in transcript level at DAFB30 in response to GA_3_ treatment, which is consistent with a previous report that GA does not influence *KNAT* promoter activity (Truernit et al., 2006). The transcription of *MdKNOX1/20*, *MdKNOX4/12*, *MdKNOX7*, *MdKNOX8*, *MdKNOX10/22*, *MdKNOX15*, *MdKNOX16*, *MdKNOX18*, and *MdKNOX19* was inhibited at DAFB50 (**Figure 7**). These results suggested that GA_3_ inhibited floral induction in apple, which might be partly achieved by inhibiting the expression of *MdKNOX* genes. Environmental factors, such as drought (which is common on the Loess Plateau), stimulates ABA accumulation and triggers an early flowering response (Verslues and Juenger, 2011). Only *MdKNOX4/12*, *MdKNOX7*, *MdKNOX10/22*, and *MdKNOX15* were induced by exogenous ABA treatment. This was especially the case for *MdKNOX4*/*12*, for which the transcript level increased over time in response to ABA treatment. Exogenous SA treatment induces accumulation of SA and accelerates the transition to flowering (MartãNez et al., 2004). *MdKNOX18* was induced by exogenous SA at 30 DAFB, whereas all other *MdKNOX* genes were not affected. The transcript levels of *MdKNOX15* and *MdKNOX19* were significantly suppressed at the intermediate stage of flower induction (50 DAFB) (**Figure 8**).

### *MdKNOX* Gene Under the Transcriptional Regulation of *MdGRF*

*KNOX* gene expression is regulated at multiple levels to prevent misexpression. Several regulators of *KNOX* gene expression have been identified, including MYB domain transcription factors (Waites et al., 1998), *ASYMETRIC LEAVES1* (*AS1*) (Byrne et al., 2002), *CUP-SHAPED COTYLEDON* (*CUC*) (Hibara et al., 2003), and *GROWTH-REGULATING FACTOR* (*GRF*) (Kuijt et al., 2014), *YABBY* (Kumaran et al., 2002), and *FIE* and *CLF* (Katz et al., 2004). The plant-specific *GRF* transcription factors, which are negative regulators of *KNOX* genes, were identified for their roles in developmental processes, including root, stem, and leaf development, flower and seed formation, and coordination of growth processes under adverse environmental conditions (Omidbakhshfard et al., 2015). The *MdGRF* genes in the apple genome were identified in previous work in our laboratory (Zheng et al., 2018). The *MdGRF* gene (MD00G1142400) used in the present research was homologous to *Arabidopsis AtGRF5* (full-length sequence identity was 30%, and the characteristic WRC and QLQ domains were highly conserved), and showed a negative correlation with *MdKNOX15* and *MdKNOX19* at the transcript level in transcriptome data (data not shown). Therefore, the interaction between *MdKNOX* and *MdGR* was evaluated in the current study. As a transcriptional regulator, MdGRF protein could direct localization of the GFP marker protein to the nucleus (**Supplementary Figure 2**). As shown in **Figure 10**, the promoter regions of *MdKNOX15* and *MdKNOX19* contained the putative *GRF* binding sites, core ‘CAG' repeats or its reverse complementary sequence ‘CTG' (Kuijt et al., 2014). Therefore, *MdKNOX15* and *MdKNOX19* were chosen to detect the interaction. MdGRF protein interacted with the *MdKNOX15* and *MdKNOX19* promoter in yeast. In rice, *KNOX* gene expression is down-regulated by *GRF* overexpression and is up-regulated by RNA interference (RNAi)-mediated *GRF* silencing. In the present study, the promoter activities of *MdKNOX15* and *MdKNOX19* were inhibited by *MdGRF* (**Figure 11**). Taken together, these results suggest that *GRF*–*KNOX* interactions might be conserved both in herbaceous and woody plants.

## Conclusion

Twenty-two *KNOX* genes were identified in the apple (*M. domestica*) genome. The *MdKNOX* members were divided into three subfamilies based on their phylogenetic relationships. Duplications have likely been important for the expansion and evolution of *MdKNOX* genes. The majority of *MdKNOX* genes exhibit strongly preferential expression in floral buds and are significantly up-regulated during the flower induction period, implying that they perform specific roles in floral induction. Furthermore, most *MdKNOX* genes are responsive to flowering-related and stress-related hormone treatments, suggesting that the genes are involved in flowering and stress response processes. The putative upstream regulatory factor *MdGRF* is able to bind directly to the promoter of *MdKNOX15* and *MdKNOX19*, and inhibits their transcriptional activities, as confirmed by Y1H and dual-luciferase assays. To our knowledge, this study is the first systematic and in-depth analysis of apple *KNOX* genes. The data provide useful information for future functional characterization of apple *KNOX* genes.

## Data Availability Statement

All datasets generated for this study are included in the article/**Supplementary Material**.

## Author Contributions

PJ, XR, and MH designed the experiments and analyzed the data. PJ, CZ, LX, XZ, YL, and KS collected the sample materials and completed the field and laboratory measurements. PJ, DZ, NA, and XR wrote the manuscript. All authors have read and approved the manuscript.

## Funding

This study was sponsored by the Chinese Postdoctoral Science Foundation (2018M631207), the Ecological Adaptability Selection of Apple Superior Stock and Scion Combinations in the Loess Plateau (A2990215082), the Screening and Interaction Molecular Mechanism of Apple Stock and Scion Combinations (K3380217027), and the National Apple Industry Technology System of the Agriculture Ministry of China (CARS-27).

## Conflict of Interest

The authors declare that the research was conducted in the absence of any commercial or financial relationships that could be construed as a potential conflict of interest.

## Supplementary Material

Supplementary Figure 1 Predicted dimensional structures of MdKNOX proteins.

Supplementary Figure 2 Subcellular localization assays of MdGRF in Nicotiana benthamiana leaves. Leaves from 5-week-old tobacco plants were infiltrated with Agrobacterium strain GV3103 harboring the 35S:GRF-GFP or 35S:GFP expression cassette, as described in section 2.7. DAPI (4',6-diamidino-2-phenylindole) was used to stain the nucleus. Green fluorescent protein (GFP) signals in transformed tobacco leaves were detected with a LEICA TCS SP8 confocal microscope (Wetzlar, Germany).

## Abbreviations

KNOX, Knotted1-like Homeobox; GRF, Growth-regulating factor; 6-BA, 6-Benzylaminopurine; GA_3_, gibberellic acid; ABA, abscisic acid; SA, salicylic acid; AbA, Aureobasidin A; DAFB, days after full bloom.
