# Supplementary figures and images for: Genome-Wide Identification of the MdKNOX Gene Family and Characterization of Its Transcriptional Regulation in Malus domestica
Source: Front Plant Sci. 2020 Feb 21;11:128. doi: 10.3389/fpls.2020.00128 (PMC7047289; doi:10.3389/fpls.2020.00128)

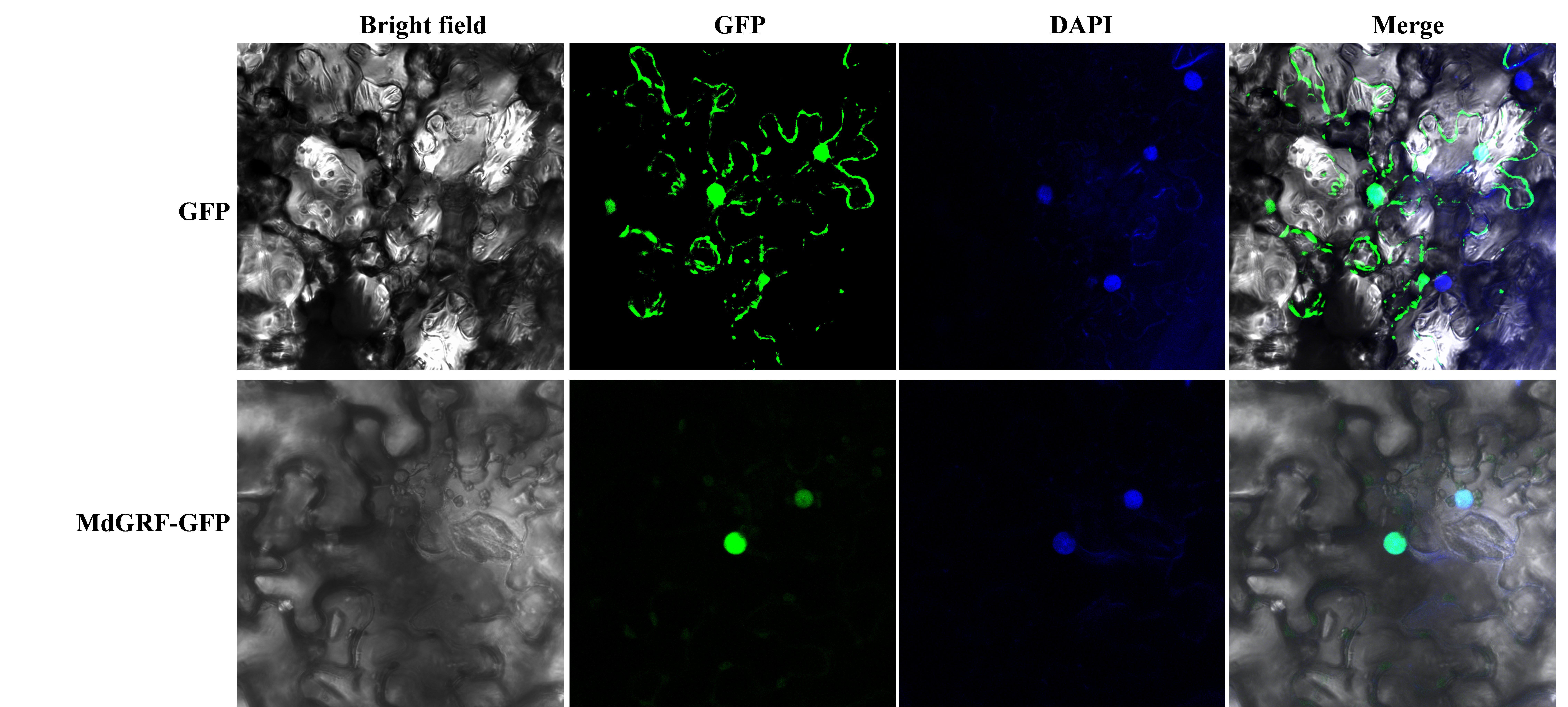

Supplement: Supplementary Figure 2 — Subcellular localization assays of MdGRF in Nicotiana benthamiana leaves. Leaves from 5-week-old tobacco plants were infiltrated with Agrobacterium strain GV3103 harboring the 35S:GRF-GFP or 35S:GFP expression cassette, as described in section 2.7. DAPI (4',6-diamidino-2-phenylindole) was used to stain the nucleus. Green fluorescent protein (GFP) signals in transformed tobacco leaves were detected with a LEICA TCS SP8 confocal microscope (Wetzlar, Germany). [file Image_2.tif]
