# Supplementary material for: Genome-Wide Identification of the MdKNOX Gene Family and Characterization of Its Transcriptional Regulation in Malus domestica
Source: Front Plant Sci. 2020 Feb 21;11:128. doi: 10.3389/fpls.2020.00128 (PMC7047289; doi:10.3389/fpls.2020.00128)
Supplement: Supplementary file 3 [file Table_1.docx]

Supp. Table 1 Primers used in this study.

| **Primer name** | **Oligo sequences (5′→3′)** |
| --- | --- |
| KNOX1/20-F | CGATGATGATGACCAGTTAGATAGCGACAT |
| KNOX1/20-R | CCAGTTCATGCTTCAGTTCCTGCCTTA |
| KNOX2/5-F | AACACCATAACCACCGTTGGATC |
| KNOX2/5-R | CCTCTCACATGTAATTAGTTGGCCAT |
| KNOX3-F | CGGTGGTGCTCATTCTAGTGGTGAT |
| KNOX3-R | GGTAGTCCTGGCATCCTTCGGTAG |
| KNOX4/12-F | AGCAGCAGTAATGGAGGAGGAATGTG |
| KNOX4/12-R | AGGTTGATGATGATGATGATGATGGTGAGA |
| KNOX6-F | AGGTGGACTACTACGACAACTACAG |
| KNOX6-R | CGAAGCAGCGTCTGACACTC |
| KNOX7-F | GTGCTTGGACATTCTGACCTC |
| KNOX7-R | GTTGATCAGGCAGATGGGAGT |
| KNOX8-F | GCTTCACTTCCTCGCCTCCAT |
| KNOX8-R | CAAACCACGACGACCAGAGT |
| KNOX9-F | GGTATACCAATTGAACCATTCATTCGTCTTAAG |
| KNOX9-R | ACATCTCGACTGGCTCTCATTTCCT |
| KNOX10/22-F | TCGCCTCTTGTCTGCCTATGTCAATT |
| KNOX10/22-R | CTCCTCCTCCTCTTATGCTACTCCTCTT |
| KNOX11-F | AAGCACCATCGTCTTCACTGTTCCTC |
| KNOX11-R | GTCATTGATCGCATTAGCCTCTTCTGGA |
| KNOX13-F | GATGAAGCAACCACATTCTTGACCAA |
| KNOX13-R | GAACGAGCACCACCAGTGTC |
| KNOX14-F | ATGGTGGGTTTGGGTGAAATTGGAGAT |
| KNOX14-R | GCTGCTGGTAGCGTCCTTGATGAAT |
| KNOX15-F | AGCAGCAACAGCAGCAGAAG |
| KNOX15-R | GTAATCCAGCGGCAGTATCAGAT |
| KNOX16-F | TCTTTGTGTCTTTCCCAGTTTCCCTAAGC |
| KNOX16-R | GCATAGCAGCCATTGACGAAGAGTAGTAA |
| KNOX17-F | TTGGACTGCTTGAAGGTAAGTGGAATAATC |
| KNOX17-R | CCATGAAGTGATCGAGGTCAGATTGTC |
| KNOX18-F | TCTTCAGTCAAGCGGCGGTGGA |
| KNOX18-R | GTTGTGATGTGGTGGCTTCGGTCTT |
| KNOX19-F | AGCATAGGACTTCATGGAAGAAGACT |
| KNOX19-R | AATCGCACCTTCACAATCAGAGTTA |
| KNOX21-F | TTCTTCATCATCTGCTCGTGTCTCTTCC |
| KNOX21-R | AACCTACGCATAAGCCGATCCTTGAG |
| Actin-F | CCATTGGAGCAGAGCGTTTCC |
| Actin-R | GGAACATGGTCGAACCACCACTA |
| Histone-F | ATGGCCCGTACCAAGCAAACT |
| Histone-R | GTACTTACGGATTTCACGAAGAGCAAC |
| MdGRF-F1 | gccatggaggccagtgaattcAATAGCACTGGAAGAAACAGCCGGT |
| MdGRF-R1 | cagctcgagctcgatggatccTCAGTCATTATTATGATGTCTTGAGGTGAAG |
| proKNOX15-F | CATTCCTCCACTACGCGTGA |
| proKNOX15-R | CAACGTATACAACCCCACAAGC |
| proKNOX19-F | ACGCTGGCTAGGATGACGTGG |
| proKNOX19-R | GAATACGGATGATCAGCTGGGTACAC |
| proKNOX15-AbAi-F | agcacatgcctcgaggtcgacTGCAGTTACCCTCTGCCACT |
| proKNOX15-AbAi-R | gaaaagcttgaattcgagctcCAACGTATACAACCCCACAAGC |
| proKNOX19-AbAi-F | agcacatgcctcgaggtcgacTTTGCCCTCCCCGAAAACTAC |
| proKNOX19-AbAi-R | gaaaagcttgaattcgagctcGAATACGGATGATCAGCTGGGTACAC |
| MdGRF-F2 | gttcttcactgttgatacatatgAATAGCACTGGAAGAAACAGCCGGT |
| MdGRF-R2 | gaattcggatccggtaccGTCATTATTATGATGTCTTGAGGTGAAG |
| proKNOX15-Dluc-F | ctgcaggtcgacggatccccgggCATTCCTCCACTACGCGTGA |
| proKNOX15-Dluc-R | ggtggactcctcttagaattcCAACGTATACAACCCCACAAGC |
| proKNOX19-Dluc-F | ctgcaggtcgacggatccccgggACGCTGGCTAGGATGACGTGG |
| proKNOX19-Dluc-R | ggtggactcctcttagaattcGAATACGGATGATCAGCTGGGTACAC |
